# Oxygen Toxicity to the Immature Lung—Part II: The Unmet Clinical Need for Causal Therapy

**DOI:** 10.3390/ijms221910694

**Published:** 2021-10-02

**Authors:** Judith Behnke, Constanze M. Dippel, Yesi Choi, Lisa Rekers, Annesuse Schmidt, Tina Lauer, Ying Dong, Jonas Behnke, Klaus-Peter Zimmer, Saverio Bellusci, Harald Ehrhardt

**Affiliations:** 1 Department of General Pediatrics and Neonatology, Justus-Liebig-University, Universities of Giessen and Marburg Lung Center (UGMLC), Member of the German Lung Research Center (DZL), Feulgenstrasse 12, 35392 Giessen, Germany; judith.behnke@paediat.med.uni-giessen.de (J.B.); Constanze.M.Dippel@med.uni-giessen.de (C.M.D.); Yesi.Choi@med.uni-giessen.de (Y.C.); Lisa.Rekers@med.uni-giessen.de (L.R.); Annesuse.Schmidt@paediat.med.uni-giessen.de (A.S.); tina.lauer@paediat.med.uni-giessen.de (T.L.); Ying.Dong@paediat.med.uni-giessen.de (Y.D.); klaus-peter.zimmer@paediat.med.uni-giessen.de (K.-P.Z.); 2 Department of Internal Medicine V, Justus-Liebig-University, Universities of Giessen and Marburg Lung Center (UGMLC), Member of the German Lung Research Center (DZL), Klinikstrasse 33, 35392 Giessen, Germany; jonas.behnke@innere.med.uni-giessen.de; 3 Department of Internal Medicine II, Universities of Giessen and Marburg Lung Center (UGMLC), Cardiopulmonary Institute (CPI), Member of the German Center for Lung Research (DZL), Justus-Liebig-University, Aulweg 130, 35392 Giessen, Germany; saverio.bellusci@innere.med.uni-giessen.de

**Keywords:** chronic lung disease, bronchopulmonary dysplasia, preterm, reactive oxygen species, lung development, inflammation, lung injury, antioxidant therapy, anti-inflammatory therapy

## Abstract

Oxygen toxicity continues to be one of the inevitable injuries to the immature lung. Reactive oxygen species (ROS) production is the initial step leading to lung injury and, subsequently, the development of bronchopulmonary dysplasia (BPD). Today, BPD remains the most important disease burden following preterm delivery and results in life-long restrictions in lung function and further important health sequelae. Despite the tremendous progress in the pathomechanistic understanding derived from preclinical models, the clinical needs for preventive or curative therapies remain unmet. This review summarizes the clinical progress on guiding oxygen delivery to the preterm infant and elaborates future directions of research that need to take into account both hyperoxia and hypoxia as ROS sources and BPD drivers. Many strategies have been tested within clinical trials based on the mechanistic understanding of ROS actions, but most have failed to prove efficacy. The majority of these studies were tested in an era before the latest modes of non-invasive respiratory support and surfactant application were introduced or were not appropriately powered. A comprehensive re-evaluation of enzymatic, antioxidant, and anti-inflammatory therapies to prevent ROS injury is therefore indispensable. Strategies will only succeed if they are applied in a timely and vigorous manner and with the appropriate outcome measures.

## 1. The Global Disease Burden of Bronchopulmonary Dysplasia after Preterm Delivery

Bronchopulmonary dysplasia (BPD) constitutes one of the most frequent and severe sequelae following premature delivery and is characterized by the distortion of physiologic lung development. From epidemiologic studies, the dominant risk factors for BPD are immaturity and very low birth weight. Further variables, including the male gender, intrauterine growth restriction, maternal nicotine abuse, chorioamnionitis, and genetic disposition, need to be considered when judging the risk of BPD [1]. Furthermore, the immature lung is an organ under development. Recent studies have clearly pointed out that adequate nutritional supply after birth is a prerogative for proper lung development [2]. Studies in other inflammatory disease entities have documented the potent modulatory properties of bacteria-derived factors from beneficial strains that need to be studied in this context [3]. The wide variations in BPD rates between different centers and regions in the world cannot be solely explained by the genetic background. Rather, discrepancies in perinatal and postnatal management need to be considered, including antenatal steroids, ventilation strategies, surfactant replacement, nutritional management, feeding with breast milk, postnatal infections, and antibiotic exposure. The latter three highly impact the patterns of microbial colonization, and recent studies have indicated an unfavorable impact of the long-term persistence of disrupted microbiota on the pulmonary outcome of preterm infants [4,5]. From the actual meta-analyses on antenatal steroids and surfactant therapy, it becomes clear that even therapies significantly contributing to respiratory stability after birth cannot reduce the burden of BPD [6,7]. The particular importance of BPD arises from the follow-up studies in former preterm infants. They documented that catch-up growth of the lung during infancy cannot be obtained, leading to lifelong restrictions in lung function. These are present not only in infants with the diagnosis of BPD but also in all infants born with extremely low birth weight and even in preterm infants of higher gestation, although to a lesser extent. Despite a lack of lifetime data for the generation of preterm infants followed-up in actual cohort studies, the risks for early-onset of a chronic obstructive pulmonary disease (COPD)-like lung disease in adulthood constitute a realistic scenario. Furthermore, the restrictions in lung function pose a high risk to the preterm infants for abnormalities in somatic growth and psychomotor development, with tremendous additional costs for the socio-economic system [8]. 

Despite all advances in the management of preterm infants in the last decades, the prevalence of BPD did not decline, which cannot be solely attributed to the higher survival rates of the most immature infants that are at particular risk for BPD. One of the imminent threats to the lung is the exposure of the preterm infant to a hyperoxic environment after birth, resulting in higher arterial oxygen tensions than that in utero. While the fetus is, in utero, provided with nutritional supply and protected from oxidative stress by its mother, the preterm infant needs to take over these tasks on his own in a situation of particular vulnerability. In the last decades, manifold research approaches were undertaken to understand the lung pathologies arising from oxygen toxicity and search for preventive or therapeutic strategies to protect the immature lung. Within the first part of our review, we focused on the pathomechanistic insights and promising therapeutic approaches in rodent models [9]. There, we provided detailed mechanistic insights, including figure illustrations. It has been demonstrated in these models that even short periods of hyperoxia exposure of 30 min can have long-term effects on lung histology [10]. This second part of our review series is dedicated to summarizing the current knowledge of the consequences of oxygen exposure to preterm infants during their NICU stay. The successful therapeutic application in rodent models of hyperoxia contravenes the disappointing low or absent efficacy in clinical trials in preterm infants. We discuss potential reasons for these disparities and novel approaches that might be suited to overcome this gap.

## 2. Oxygen Requirements of the Preterm Infant

Postnatally, the preterm infant is exposed to relative hyperoxia reaching 60–100 mmHg to meet oxygen saturation goals, compared to its intrauterine environment with 25–30 mmHg of arterial oxygen pressures [11]. After birth, the preterm infants’ poorly developed defense mechanisms against hyperoxia get shortly overwhelmed by reactive oxygen species (ROS) release. Further sources of ROS arise from accompanying perinatal infections, birth asphyxia, and, i.e., increased levels of free iron. It is not surprising that the cumulative oxygen exposure during the first 72 h of life is highly predictive for the pulmonary outcome and BPD [12]. 

Despite this particular vulnerability, a randomized clinical trial in preterm infants <32 weeks did not demonstrate differences in psychomotor outcome when the infants were resuscitated with room air or 100% oxygen [13]. Of note, the posthoc analysis demonstrated that reaching an oxygen saturation level above 80% at 5 min after birth was associated with a better outcome regarding survival and psychomotor outcome at 2 years of corrected age [13,14]. This is corroborated by further observation that lower oxygen saturation levels at 5 min after birth are associated with lower cerebral oxygenation [15]. A posthoc analysis of infants born below 28 weeks of gestation revealed 3-fold higher mortality in those infants that were stabilized with room air in the delivery room compared to infants provided with 100% oxygen [13]. In contrast, another study with a similar approach revealed higher levels of oxidized glutathione in the peripheral blood; urinary markers of oxidative stress were increased in the infants provided with higher oxygen fractions in the delivery room, and these infants had a higher risk of prolonged oxygen use, need of mechanical ventilation, and BPD [16]. Similarly, another study detected better preserved antioxidative capacities and lower hydroperoxides shortly after birth when a strategy of limited oxygen application was pursued in the delivery room [17]. These data were reproduced in a similarly structured study, while a third study did not detect any statistically significant difference [17,18]. The recent meta-analysis, pooling altogether all eight published studies using lower (≤30%) versus higher (≥60%) fractions of oxygen in the delivery room, aimed to resolve these discrepancies. It revealed lower heart rates and an increased risk for intraventricular hemorrhage in the lower fraction group. Not reaching SpO2 levels above 80% at 5 min of age was associated with worse outcomes; however, it remains still unclear whether the increased risk is due to the higher disease severity of infants or the lower amount of oxygen applied. From the available clinical datasets, no increased risk for BPD can be deducted by the provision of higher levels of oxygen after birth, but as only 12% of infants reached the oxygen saturation target, no valid conclusion can be drawn so far [19]. It must be stated from the published studies that the disparity of starting with lower or higher fractions of oxygen is restricted to the first minutes of life as both arms ended up with comparable fractions of oxygen required to meet oxygen saturation targets at 10 min of life [15,16,17]. One recent randomized pilot study investigated the feasibility of combining oxygen supply with inhalative NO during the first 20 min of life in the delivery room. In the intervention arm, the cumulative FiO_2_ exposure and the duration of exposure to oxygen fractions above 60% were reduced [20]. This study can be considered a starting point to investigate novel strategies to reduce the oxidative burden after birth.

Beyond the delivery room, several randomized multicenter studies compared different narrow saturation levels during the stay in the neonatal ward in the last two decades and delivered identical results: while oxygen saturation levels above 95% increase the risk for BPD, a saturation range between 85% and 89% leads to increased mortality before discharge and rates of necrotizing enterocolitis; in contrast, the risk for retinopathy of prematurity and BPD is reduced when the BPD definition is based on persisting oxygen dependency at 36 weeks of gestation [21,22,23]. The extension of this meta-analysis to the 24-month follow-up failed to prove a benefit of higher oxygen saturation limits (90% to 95%) for the composite outcome of mortality or major disability and major disability alone [22]. 

Actual recommendations from the European consensus guideline are based on these results and recommend using 30% oxygen in the delivery room for infants <28 weeks of gestation; neonatologists are recommended to meet an oxygen saturation target of 80% and a heart rate above 100/min at 5 min after birth [24]. The latest consensus paper of the AAP on oxygen saturation targeting is more reserved but states that a corridor between 91% and 95% is currently safer. However, it includes a statement that there still remain major uncertainties about the optimal range for extremely low birth weight infants [25]. Of note, all these trials did not change their oxygen targeting approach throughout the postnatal course. Hence, no data are available on whether preterm infants can benefit from different oxygen target corridors at different stages postnatally. Further limitations of these studies were the reported selection bias and that oxygen targeting was not set into the context of lung aeration of what was not feasible with the available techniques [26]. Sustained inflation maneuvers, aiming to aerate the lung immediately after birth, have been shown to be associated with an increased risk of death within the first 48 h, and no benefit on the outcome of death or BPD at a corrected age of 36 weeks of gestation was seen [27]. However, even after almost 50 years of CPAP therapy in the treatment of preterm infants, the optimal PEEP in the delivery room and during acute respiratory distress in the days after birth to efficiently aerate the lung is not clear. Hopefully, the just-started OPTTIMMAL study will provide new insights on this topic [28]. Experimental data consistently indicate that lung injury by hyperoxia is reduced in the presence of CPAP, underlining the potential of optimizing lung aeration to prevent ROS-induced lung injury [29,30]. Whether the reduction of atelectasis and resulting lung injury in the absence of PEEP accounts for the increase in inflammation and lung injury remains elusive due to limitations in the size of rodent studies. Whether more sophisticated techniques such as synchronized NIPPV provide further advantages in the context of oxygen toxicity or primarily benefit the lung by reducing the risk of intubation remains to be determined [31]. Results from murine models indicate that the duration and extent of oxygen exposure constitute the decisive variables responsible for the mechanisms and extent of lung injury [32]. However, it remains unclear whether this holds true for the situation of the preterm infant in the NICU. Currently, it is unclear how gradual weaning from oxygen impacts lung histology and function [33].

One further dimension arises from the scientific knowledge: not only hyperoxia per se but intermittent hypoxic episodes followed by reoxygenation contribute to ROS production. The principal molecular mechanisms and the current state of knowledge on the topic underlying this particular vulnerability have been summarized recently [34]. One pioneering posthoc analysis from the oxygen-saturation-targeting COT trial confirmed that prolonged hypoxic episodes were associated with an increased risk for death or disability at the 19-month follow-up. Available clinical data suggest comparable mechanisms of lung, eye, and brain injury in the context of ROS [35,36]. Experimental data provided important insights into the aggravation of lung injury by hyperoxia in combination with or followed by repeated hypoxic episodes. Both situations, which are frequently present in preterm infants, contribute to the aggravation of hyperoxic lung injury [37,38]. In further studies in rats, only hyperoxia with intermittent hypoxia decreased the antioxidative capacities in the lung and lung injury. The inhibition of ROS preceded the influx of inflammatory cells and prohibited lung injury [37,39]. These data allow the conclusion that approaches towards preventing ROS-induced lung injury need to take into account both hyperoxia and hypoxemia and that these new models combining both clinical features in preterm infants better reflect the pathogenesis of BPD. It must be assumed that reoxygenation injury exceeds ROS production and lung injury provoked by hyperoxia alone [37]. The two observational clinical studies on hypoxemic episodes and the occurrence of BPD found an association with the number of hypoxemic episodes within the first month of life and the probability of BPD [40,41]. A recent posthoc analysis of extremely preterm infants from the aforementioned COT trial, investigating prolonged intermittent hypoxemia beginning in the first week after birth, showed an association with an increased risk of developing severe BPD [42]. Due to the observational character of these studies, the mechanistic link between hypoxemia and BPD is not clear. Either the early alterations in lung function with, i.e., reduced functional residual capacity are a precedent of BPD and account for these desaturations or the hypoxemic episodes per se contribute to BPD. Actually, the available data do not allow a conclusion as to what depth and extent of hypoxic episodes are dangerous to the immature lung. 

## 3. ROS-Induced Injury to the Immature Lung as a Key Driver of BPD

Increased arterial oxygen tension after birth and the application of high concentrations of oxygen to the immature lung initiate a cascade of ROS production, mainly superoxide, hydrogen peroxide, hydroxyl radicals, and peroxynitrite. Mitochondria are the main source of ROS, but peroxidases, lipoxidases and myeloperoxidases, nicotinamide adenine dinucleotide phosphate (NADPH) and xanthine oxidation, superoxide anions, glutathione peroxidation, cyclooxygenases, lipoxygenases, and reactive nitrogen species contribute to the free radical overload while NO synthesis is decreased [43]. These free radicals elicit direct toxicity to the lung and initiate further downstream cascades, aggravating lung damage. The intimate crosstalk between epithelium, mesenchyme, and endothelium in the lung explains why ROS can induce such dramatic consequences to all compartments of the lung. In this context, the hyperoxia-mediated degradation of HIF1α and subsequent suppression of vascular endothelial growth factor (VEGFA) signaling constitutes a key event following oxygen exposure. The successful preclinical prevention of vascular and alveolar growth compromise by VEGFA-targeted therapies has proved the high therapeutic potential of strategies targeting hyperoxic injury [44,45,46]. The hallmarks of ROS-induced injury are subsequently detailed and form the basis for the clinical evaluation of therapies aiming to prevent oxidative injury. 

### 3.1. Underdeveloped ROS Defense Mechanisms

Defense mechanisms against relative hypoxia are already well-established in utero and are mainly regulated via HIF1α signaling that can activate several hundred downstream genes that are partly and crucially involved in the defense to hypoxia. In contrast, the antioxidative enzymes and non-enzyme oxidative system are underdeveloped until shortly before term delivery. Furthermore, maternal transfer of non-enzymatic antioxidants via the placenta is established only shortly before birth. Antioxidative capacity is constituted by superoxide dismutases, catalases, heme oxygenases, flavin-containing enzymes, glutathione, and thioredoxin, which are the most prominent and best-studied candidates. This makes preterm infants especially vulnerable to ROS-induced injury [47]. The most relevant features of ROS tissue injury, following their accumulation, are ascribed to oxidative damage to proteins, lipids, and DNA [48]. Data in rodents showed profound mitochondrial oxidant stress while antioxidant counter-regulation as a defense mechanism was absent [49]. The vicious circle is reinforced as damaged mitochondria are no longer available for oxidative phosphorylation. Clinical data in human umbilical vein endothelial cells (HUVECs), obtained from preterm infants at birth, confirm this assumption. Cells from infants that later developed BPD or died displayed mitochondrial dysfunction with lower oxygen consumption but increased oxidant production [50]. 

### 3.2. Cell Death Induction

One of the dominant actions of ROS is to induce cell death in the lung. Epithelial cells seem particularly vulnerable, and experimental data have provided conclusive evidence that mitochondrial oxidation, as the key source of ROS, initiates mitochondrial pore formation and subsequent cell death induction [51].

### 3.3. Inflammation

ROS induce a pro-inflammatory response in the lung and contribute to the attraction of inflammatory leukocytes. Activated macrophages constitute a major source of pro-inflammatory cytokines. While ROS production of neutrophils constitutes an important mechanism of bactericidal defense, it can also lead to self-injury. Furthermore, neutrophils release plenty of proteases that aggravate the hallmarks of lung injury and inactivate surfactants [52]. In conclusion, the downstream inflammatory response in the lung constitutes a key driver of the aggravation of ROS production and lung injury.

### 3.4. Epigenetic Modifications 

In addition to the direct effects of ROS on protein production and metabolism, epigenetic regulation is one further dimension with potential long-term consequences for normal and abnormal lung development and growth with a high concordance between mice and men [53]. DNA methylation and histone modification of signaling pathways involved in inflammation, growth, and repair are implicated in the context of BPD [54]. Clinical studies have clearly demonstrated a connection between the oxygen load provided and the extent of DNA hypermethylation. These mechanisms become evident already shortly after birth when the preterm infant is exposed to higher fractions of oxygen, demonstrating its particular vulnerability. The long-term consequences of these changes in DNA methylation need to be specified in future studies. These persistent long-term changes in the lungs of former preterm infants just recently became evident and await further exploitations. 

The evidence from preclinical studies is convincing that oxygen toxicity has acute effects on the lung via ROS, while long-term consequences can be ascribed to epigenetic gene silencing [55]. 

## 4. Clinical Approaches to Prevent ROS-Induced Lung Injury and BPD

The convincing data from preclinical studies of mostly rodent and lamb models, summarized in the first part of our review series where ROS-induced injury to the immature lung and the development of BPD can be prevented by antioxidative therapies, were in contrast to their investigation in clinical randomized trials, where they mostly failed to prove therapeutic efficacy [9]. Next, we summarize the available data (detailed in Table 1, Table 2 and Table 3) andd search for explanations for the gap between preclinical efficacy and missing clinicalproof of benefit:

### 4.1. Antioxidants

The best-studied antioxidant in BPD is vitamin A. Although its efficacy is limited and restricted to the intramuscular route of administration, it is the only antioxidant drug, so far, with proven effect to reduce the incidence of BPD [56]. The data from vitamin A substitution trials in the preterm infant clearly indicate the necessity of a careful investigation of the consequences of antioxidant substitution on plasma levels, downstream targets, and antioxidative capacities to document the successful elevation of antioxidative capacities [57]. Data from one trial of oral vitamin A substitution versus placebo indicate the principal efficacy of vitamin A when given orally [58]. The ongoing NeoVitaA multicenter study, with oral applications of six-fold of the recommended dosage in the intervention group, is powered to clarify the benefits of oral vitamin A substitution [59]. One actually published smaller-sized study using enteral water-soluble vitamin A documented no benefit for BPD, while plasma retinol levels were significantly elevated with a near doubling in the intervention group [60]. As for vitamin A, vitamin E deficiency shortly after birth was repeatedly associated with increased pulmonary morbidity [61]. However, the substitution of vitamin E under the monitoring of plasma levels did not prevail any benefit for the lung in infants with a birth weight <1500 g. This was confirmed in the meta-analyses of 7 studies [62,63]. Much hope was set into glutathione and its precursors to prevent BPD. Sequential measurements in preterm infants revealed a deficiency that can compromise the lungs´ defense mechanisms against oxidative stress and is associated with BPD [48,64]. However, the approach to overcome postnatal glutathione deficiency in the preterm infant by substitution with n-acetylcysteine (NAC), a derivate of the amino acid cysteine (one of three essential peptides for glutathione synthesis), for 6 days after birth did not reduce the risk of BPD or death and did not improve lung function measures at term equivalent age [65,66]. Lastly, docosahexaenoic acid (DHA), an n-3 long-chain polyunsaturated fatty acid, was tested within a multicenter trial to prevent BPD due to its antioxidant capacities. In contrast to the expectations and results from previous smaller studies, the risk of BPD was not reduced [67]. Further, ROS scavengers such as ascorbic acid and astragaloside show promising preclinical results, but results from clinical studies are still missing.

Finally, caffeine as a milestone drug in modern neonatal healthcare, with proven efficacy to prevent BPD, has also been demonstrated to possess antioxidant activity. Its main action in the clinical setting is currently attributed to stabilizing the respiratory drive of the preterm infant, but its further activities include diuretic and anti-inflammatory properties. For the last-mentioned action, convincing experimental data proved a reduction in inflammatory injury [68]. The actual meta-analysis proves that higher dosages of caffeine are more effective in preventing BPD. This finding must be seen in the context that higher dosages reduce the duration of mechanical ventilation and more efficiently prevent reintubation [69]. The significant differences deserve further efforts to clarify the optimal dose, as is currently pursued for vitamin A.

Lastly, nitric oxide needs to be discussed in this section. It was primarily studied as a potent vasodilator and lung-growth-promoting gas, supplied within the inspiratory gas during the first weeks after birth. In theory, it further possesses potent antioxidant capacities. However, so far, no benefit for the outcome BPD has been detected. One pilot study indicated that there might be use for nitric oxide in the delivery room to reduce the exposure to oxygen, but this warrants further and larger-scaled studies before a recommendation can be made [20]. The results of meta-analyses showed no evidence for iNO as rescue therapy in unstable preterms, and its early routine use did not improve survival without BPD [70].

### 4.2. Enzymatic Therapy

Within the spectrum of preclinical strategies of antioxidant enzyme substitution, recombinant superoxide dismutase (rhSOD) substitution delivered the most promising results. However, the level of evidence is low. Infants requiring intubation after birth were treated during the whole course of invasive mechanical ventilation, and rhSOD was applied by intratracheal instillation. While the primary outcome of death or BPD did not demonstrate a positive effect of this intervention, follow-up at 1 year of age revealed a 36% reduction in infants with respiratory sequelae or requiring pulmonary drug treatment. The effect size was even bigger in infants below 27 weeks of gestation [71]. These contrary data on the outcome of BPD and longer pulmonary follow-up into childhood are in line with the reports from the SUPPORT trial comparing intubation and surfactant replacement therapy in the delivery room versus initial stabilization on CPAP and the UKOS HFO versus the conventional mechanical ventilation study that underlined the necessity for respiratory follow-up into childhood to dissect therapeutic benefits [72,73]. Of note, the posthoc analysis from the trial of rhSOD treatment on the incidence of ROP and severe ROP revealed a nearly 50% reduction in the group of most immature infants, which can be expected as both BPD and ROP are driven by ROS [74]. Such data raise hopes that antioxidative therapy might be beneficial for all oxygen diseases of preterm birth, including ROP, NEC, IVH, and periventricular white matter damage, which display comparable pathomechanisms such as BPD [44].

In this context, it is indispensable to discuss the antioxidative properties of natural surfactant preparations that have already been documented 30 years ago. Superoxide dismutase and catalase were retrieved from all-natural surfactant preparations in variable amounts, and intratracheal instillation in rabbits documented reabsorption into type II cells and enhanced intracellular antioxidant enzyme activity [75,76]. Subsequent studies in preterm infants confirmed that natural surfactant treatment reduced the parameters of oxidative stress in the tracheal aspirates of ventilated infants [77]. As mentioned in the introduction, the meta-analysis of surfactant replacement studies after birth did not prevail to demonstrate a therapeutic benefit for BPD [5]. As all these studies used surfactant replacement only in the direct period after birth, while premature infants are exposed to oxidative stress for long periods of time in the NICU, additional late surfactant application was suggested to be beneficial. However, so far, randomized clinical trials of late surfactant application, starting the second week of life for up to 3 doses and in combination with iNO commencing 48 h after birth for up to 5 dosages, did not show a benefit for pulmonary outcome [78,79]. Whether shorter dosing intervals or higher dosages can reverse these findings awaits proof in the future. 

### 4.3. Trace Elements as Enzyme Cofactors

Trace elements are indispensable cofactors for antioxidative enzyme function, including iron, selenium, and zinc. As preterm infants are deprived of selenium, its substitution after birth seemed particularly promising; however, despite improved plasma levels, the incidence of BPD was not reduced in a randomized trial [80]. 

### 4.4. Anti-Inflammatory Drugs

Antenatal steroids constitute one of the mainstay therapies of preterm infants, although they fail to reduce the overall BPD burden. Their main action lies in the promotion of lung maturity, thereby improving the gas exchange after birth and reducing the need for supplemental oxygen. Beneficial effects of antenatal steroid exposure on oxidative stress and protein oxidation at birth have been demonstrated in preterm infants. From the details on the timing of antenatal steroid application and interval until birth, it becomes clear that the effect of a one-time application is transient, which might contribute to the missing efficacy to reduce BPD [81]. Postnatal corticosteroid application is effective in preventing BPD due to its broad anti-inflammatory activity despite the concerns of its negative effects on further lung development and neurodevelopmental outcomes. However, a careful risk–benefit assessment is indispensable due to the side effects of postnatal steroids on psychomotor outcomes, and this therapy should be restricted to infants at high risk for severe limitations in lung function [82,83,84]. Topical corticosteroid application to the lung by inhalation did not result in an overall benefit within a multicenter randomized controlled trial. While the rate of BPD was reduced, this went along with an increased rate of death in the intervention group. The unchanged neurodevelopmental outcome at the age of 2 years might argue towards avoiding the negative effects of systemic corticosteroid application by direct deposition into the lung [85,86]. In contrast, intratracheal application in conjunction with surfactant application after birth proved their therapeutic efficacy in a first clinical study [87]. Future studies need to prove that this approach is an efficient alternative.

Vitamin D is a promising candidate due to its anti-inflammatory and lung-growth-promoting properties. Its therapeutic potential is derived from the current meta-analysis that shows that low vitamin D levels at birth predispose the infants to BPD [88]. The experimental evidence suggests a significant benefit that can, at least partly, be attributed to the inhibition of key features of the inflammatory response [89,90]. The published pilot study, comparing different vitamin D dosing regimes within the first 4 weeks after birth, was underpowered to detect a statistically significant difference, and clinical recommendations cannot be provided thereof [91]. Large, randomized trials on vitamin D are needed before any recommendation can be made.

## 5. Unmet Needs and Future Perspectives

Today, researchers around the globe still face the unmet disparities between preclinically successful therapies to prevent ROS-induced lung injury in preclinical models of BPD and the lack of benefit to preterm infants. Several reasons have been postulated. First, BPD is a multifactorial disease that arises from heterogeneous causes. Preclinical studies have mostly relied on isolated exposure to hyperoxia and the pathologies of hyperoxic injury. The scarcity of multiple-hit preclinical models has been increasingly recognized [92,93]. Second, newborn mice and rats display lung structures similar to those born very preterm but with relatively well-developed antioxidant systems that require extensive hyperoxia exposure to induce BPD-like lung injury. Third, there exists a sophisticated network of signaling pathways of lung development and lung injury. Recent insights propose that the balance of this network is a prerequisite for proper lung development that can hardly be maintained during the longitudinal course in the NICU. As suggested before, the imbalance in any direction leads to lung pathology that has been best studied for NFκB, TGFβ, HIF1α, VEGFA, and Wnt signaling. Not surprisingly, interventions targeting just one component in this complex system might not necessarily result in improved pulmonary outcomes even when the critical step of hyperoxia-mediated HIF1α degradation is addressed [31,94,95,96,97]. Fourth, ROS production originates from specific cellular structures and compartments, and ROS therapeutics need to penetrate these sites. Furthermore, a certain level of ROS is required for physiologic signaling pathway activation in the developing lung, and rigorous scavenging may interfere here. Fifth, clinical studies are hampered by the wide variation in BPD definition. The actual studies constitute cross-sectional analyses of infants born at different gestational ages, and different stages of lung development and oxygen dependency are just one parameter of gas exchange. The actual updated BPD scorings partly improve the estimation of the pulmonary outcome. Currently, lung function testing during infancy is seen as the gold standard, but it does not take into account secondary injuries to the lung by, i.e., viral infections. The documented discrepancies between BPD judgment at 36 weeks and lung function data in later childhood currently impede a timely judgment of the long-term benefit of any intervention in the period after birth, although biomarker approaches are, in principle, suited to resolve this issue [1,98,99]. Sixth, our review of the available literature clearly demonstrates that ROS production and the exhaustion of antioxidative capacities occur early after birth, especially in situations with prenatally augmented ROS production, such as amniotic infection. Therefore, the time window for successful therapeutic approaches is narrow or needs to be shifted to prior delivery. Prenatal maternal substitution or delivery of therapeutic approaches to the fetus before birth might be better suited to prove efficacy and improve the pulmonary outcome. Seventh, some of the antioxidative agents that showed efficacy in animal models might need a re-evaluation, as recently postulated for vitamin E [100]. In that case, the formulation used contained a potential harmful isoform and relied on a carrier with damaging potential to, i.e., the epithelium. Eighth, the scientific evidence and recommendations derived from meta-analyses are based, in part, on data from cohorts more than two decades ago, where preterm infants frequently had a more severe pulmonary course in the NICU and novel techniques, including synchronized non-invasive respiratory support and non-invasive surfactant application, were not established as clinical routine [101,102,103]. Our review of the clinical data on oxygen toxicity on the immature lung has, as well, several limitations. So far, only a limited number of randomized controlled trials and meta-analyses are available on this topic. In many of these studies, the primary outcome was BPD, BPD or death, or lung function during follow-up but not the impact of the therapeutic interventions on ROS production. Most of the studies dealing with ROS toxicity relied on associations; however, as in the Canadian oxygen trial, no causal link can be provided [40]. Due to the lack of data on preterm infants, we cannot provide data on complementary approaches that are available, e.g., for asthma patients [104]. Lastly, for most ROS therapies, no dose-escalation studies are available that might have concealed their efficacy.

The key to an effective solution to this dilemma is not an easy one to answer, and this is due to the complexity of BPD´s nature. ROS-driven diseases in preterm infants are caused by multiple mechanisms, which should be taken into account when developing therapeutic approaches. Furthermore, an isolated approach to one target within the complexity of BPD pathogenesis might not be sufficient to turn the tide. Considering the benefits of different approaches simultaneously might be better suited. One intensively studied topic is the therapeutic potential of exogenous MSC application to prevent BPD. Here, several different approaches, including growth-promoting, anti-inflammatory, and anti-microbial actions, are combined within one therapy [95,105]. In analogy, targeting several actions of ROS injury simultaneously might be suited to prove superiority. 

Even more than 50 years after the original description of BPD, no curative therapy is available. While the magnitude of clinical trials did not prove its therapeutic efficiency, the central role of oxygen and its deleterious effects mediated by ROS are highly recognized. Therefore, it remains essential to translate the improvements in scientific knowledge into efficient therapies. Reflecting the principles of disease initiation and disease progression and improvements in the timing and targeting of therapeutic applications and respecting the complexity of the disease and the different contributing factors can contribute to finally reaching the ultimate vision of BPD researchers.

## Figures and Tables

**Table 1:** Clinical approaches to prevent ROS-induced lung injury and BPD—antioxidants.

Intervention	Study Design	Study Population	Primary Outcome	Effect on BPD/Lung Injury	Reference
Vitamin A (supplementation vs. control, placebo, or no supplementation)	Cochrane Database Syst Rev. included trials (RCTs): 11	n = 1580 BW ≤ 1500 g or GA ≤ 32	death (at 28 days and at hospital discharge) chronic lung disease (defined as oxygen use at 28 days or 36 weeks PMA) death or chronic lung disease	small benefit in reducing the risk of chronic lung disease (RR 0.87; 95% CI 0.77–0.99)	[56]
Vitamin E (supplementation vs. either placebo, no treatment, or another type, dose, or route of administration	Cochrane Database Syst Rev. included trials (RCTs): 26	n = 2028 GA < 37 or BW < 2500 g	mortality, combined outcome at 18 months including mortality	no effect on BPD (RR 0.91; 95% CI 0.73–1.14) or mortality until discharge (RR 0.97; 95% CI 0.83–1.14)	[63]
DHA	RCT	n = 1273 GA ≤ 27	BPD (physiological basis, oxygen-saturation monitoring at 36 weeks PMA or discharge)	no effect on BPD (RR 1.13; 95% CI 1.02–1.25; *p* = 0.02)	[67]
NAC (supplementation vs. placebo)	RCT	n = 391 BW 500–999 g	death or BPD (supplementary oxygen requirement at 36 weeks PMA)	no difference in the combined incidence of death or BPD (OR 1.0; 95% CI 0.7–1.6)	[65]
Caffeine (high vs. low dose)	Meta-analysis Included trials (RCTs): 6	n = 816 GA < 34	mortality during the first admission, BPD (at 36 weeks CA), cerebral palsy	fewer cases of BPD (RR 0.76; 95% CI 0.60–0.96); quality of the evidence was low due to imprecision of the estimates	[69]
iNO (supplementation vs. control with or without placebo)	Cochrane Database Syst Rev. included trials (RCTs): 17	n = 4062	death before hospital discharge BPD (oxygen dependence at 36 weeks PMA) death or BPD (at 36 weeks PMA) IVH (any grade and more severe, grades 3 and 4)	early routine use of iNO in preterm infants with respiratory disease does not improve survival without BPD (RR 0.89; 95% CI 0.76–1.04) later use of iNO to prevent BPD could be effective, but the current 95% CI included no effect; the effect size is likely small (RR 0.92; CI 0.85–1.01) and requires further study	[70]

n—number; BW—birth weight; GA—gestational age; BPD—bronchopulmonary dysplasia; RCT—randomized controlled trial; PMA—postmenstrual age; RR—relative risk; CI—confidence interval; DHA—docosahexaenoic acid; NAC—N-acetylcysteine; iNO—Inhaled nitric oxide; IVH—intraventricular hemorrhage.

**Table 2:** Clinical approaches to prevent ROS-induced lung injury and BPD—enzymatic therapy and trace elements as enzyme cofactors.

Intervention	Study Design	Study Population	Primary Outcome	Effect on BPD/Lung Injury	Reference
r-h CuZnSOD (supplementation vs. placebo)	RCT	n = 302 BW 600–1200g GA ≥ 24	death or BPD (oxygen dependency at 28 days of life, with a chest radiograph with an Edwards score ≥3) at 28 days of life	no differences in BPD or death incidence (*p* = 0.11), treatment at birth may reduce early pulmonary injury, with improved clinical status when measured at 1-year corrected age	[71]
Surfactant (animal-derived treatment vs. control treatment in RDS infants)	Cochrane Database Syst Rev. included trials (RCTs): 13	GA < 37	neonatal mortality (mortality <28 days of age) from any cause mortality prior to hospital discharge (from any cause) BPD (oxygen requirement at 28 to 30 days of age) BPD or death prior to 28 days of age chronic lung disease (use of supplemental oxygen at 36 weeks PMA) chronic lung disease (use of supplemental oxygen at 36 weeks PMA) or death prior to 36 weeks PMA	significant decrease in BPD or death at 28 days of age (RR 0.83; 95% CI 0.77–0.90) no significant impact on BPD alone (RR 0.95; 95% CI 0.84–1.08)	[6]
iNO+surfactant vs. iNO alone	RCT	n = 511 GA < 28	survival at 36 weeks PMA without BPD (evaluated by physiological oxygen/flow reduction)	no differences on BPD at 36 weeks PMA (95% CI 0.75–1.28; *p* = 0.89)	[79]
Selenium (supplementation vs. control)	RCT	n = 534 BW < 1500g	oxygen dependency at 28 days and total days oxygen dependency	no data on BPD no effect on O_2_ dependency at 28 days (RR 0.97; 95% CI 0.80–1.18)	[80]

n—number; BW—birth weight; GA—gestational age; BPD—bronchopulmonary dysplasia; RCT—randomized controlled trial; PMA—postmenstrual age; RR—relative risk; CI—confidence interval; r-h CuZnSOD—recombinant human copper-zinc superoxide dismutase; nCPAP—nasal continuous positive airway pressure; CA—corrected age; RDS—respiratory distress syndrome.

**Table 3:** Clinical approaches to prevent ROS-induced lung injury and BPD—anti-inflammatory drugs.

Intervention	Study Design	Study Population	Primary Outcome	Effect on BPD/Lung Injury	Reference
Postnatal corticosteroids (early, <8 d) (supplementation vs. placebo or control)	Cochrane Database Syst Rev. included trials (RCTs): 32	n = 4445 GA < 37	mortality BPD (at 28 days of life, at 36 weeks PMA, and at 36 weeks PMA in survivors) death or BPD (at 28 days of life and at 36 weeks PMA) long-term outcomes	early corticosteroids reduced the incidence of BPD at 28 days of life (RR 0.87; 95% CI 0.81-0.93) and at 36 weeks PMA (RR 0.79; 95% CI 0.72–0.87)	[82]
Postnatal corticosteroids (late, >7 d) (supplementation vs. placebo or control)	Cochrane Database Syst Rev. included trials (RCTs): 21	n = 1424	chronic lung diesease (mortality and/or BPD at 36 weeks PMA)	reduction of BPD (RR 0.77; 95% CI 0.67–0.88) and combined outcome death or BPD (RR 0.77; 95% CI 0.70–0.86)	[83]
early (within 24 h after birth) inhaled budesonide (supplementation vs. placebo)	RCT	n = 863 GA > 23 < 28	death or BPD (confirmed by means of standardized oxygen-saturation monitoring at a 36 weeks PMA)	death or BPD was lower in the budesonide group, but the advantage may have been gained at the expense of increased mortality (RR 0.86; 95% CI 0.75–1.00; *p* = 0.05; OR 0.71; 95% CI 0.53–0.97)	[85]
intratracheal administration of surfactant/budesonide vs. surfactant alone	Clinical trial	n = 265 BW < 1500 g	death or BPD (supplemental oxygen at 36 weeks PMA)	lower incidence of BPD or death in intervention group (RR 0.58; 95% CI 0.44–0.77; *p* < 0.01)	[87]
Vitamin D (birth levels in BPD infants vs. control)	Meta-analysis, included trials: 8	n= 909	BPD (oxygen dependency at either 28 days of age or 36 weeks PMA)	BPD was associated with vitamin D deficiency at birth (OR 2.405; 95% CI 1.269–4.560; *p* = 0.007) and low levels at birth (standardized mean difference −1.463; 95% CI −2.900–0.027; *p* = 0.046)	[88]

n—number; BW—birth weight; GA—gestational age; BPD—bronchopulmonary dysplasia; RCT—randomized controlled trial; PMA—postmenstrual age; RR—relative risk; CI—confidence interval.
